# Transcatheter tricuspid valve replacement—a comparison of clinical and procedural outcomes of two different prostheses

**DOI:** 10.3389/fcvm.2026.1923052

**Published:** 2026-09-04

**Authors:** Florian Genske, Judith Heine, Christoph Marquetand, Thomas Stiermaier, Ingo Eitel, Christian Frerker, Tobias Schmidt

**Affiliations:** 1 Department of Cardiology, University Hospital Schleswig-Holstein, University Heart Center Lübeck, Lübeck, Germany; 2 DZHK (German Centre for Cardiovascular Research), Partner Site North, Lübeck, Germany; 3 Department of Cardiology, Asklepios Clinic Hamburg-Rissen, Hamburg, Germany

**Keywords:** cardiovalve, EVOQUE, tricuspid regurgitation, TR, Transcatheter tricuspid valve replacement, TTVR

## Abstract

**Background:**

Transcatheter Tricuspid Valve Replacement (TTVR) has emerged as a treatment option for patients with severe tricuspid regurgitation (TR) who are at high surgical risk and unsuitable for tricuspid transcatheter edge-to-edge repair (T-TEER).

**Objectives:**

This study aims to compare procedural, clinical and echocardiographic outcomes of two different TTVR devices: the Cardiovalve (Venus MedTech) and the EVOQUE (Edwards Lifesciences) prosthesis.

**Methods:**

We performed a retrospective, single-center cohort analysis including all consecutive patients who were treated with TTVR. Primary endpoints included TR reduction, NYHA class and safety events according to TVARC criteria.

**Results:**

Overall, 25 consecutive patients were included in the analysis. The Cardiovalve prosthesis was implanted in 12 patients and the EVOQUE prosthesis in 13 patients. Intraprocedural success was 100%. There was a significant reduction in TR grade (4.20 vs. 0.48; *p* < 0.001) and NYHA class (2.72 vs. 1.88; *p* = 0.004) for the overall cohort at 30-day follow-up, but no differences between the two devices (TR grade 0.50 vs. 0.46; *p* = 0.408 and NYHA class 1.75 vs. 2.00; *p* = 0.813; Cardiovalve and EVOQUE respectively). Mortality rate at 30 days was 0% in both groups. One patient in each group (8.0%) required new pacemaker implantation.

**Conclusion:**

TTVR with both, the Cardiovalve and EVOQUE prothesis is an effective treatment option, leading to profound TR reduction and meaningful clinical improvement at 30-day follow-up. Patient selection and long term follow up need to be the focus of future studies.

## Introduction

**Figure 1:**
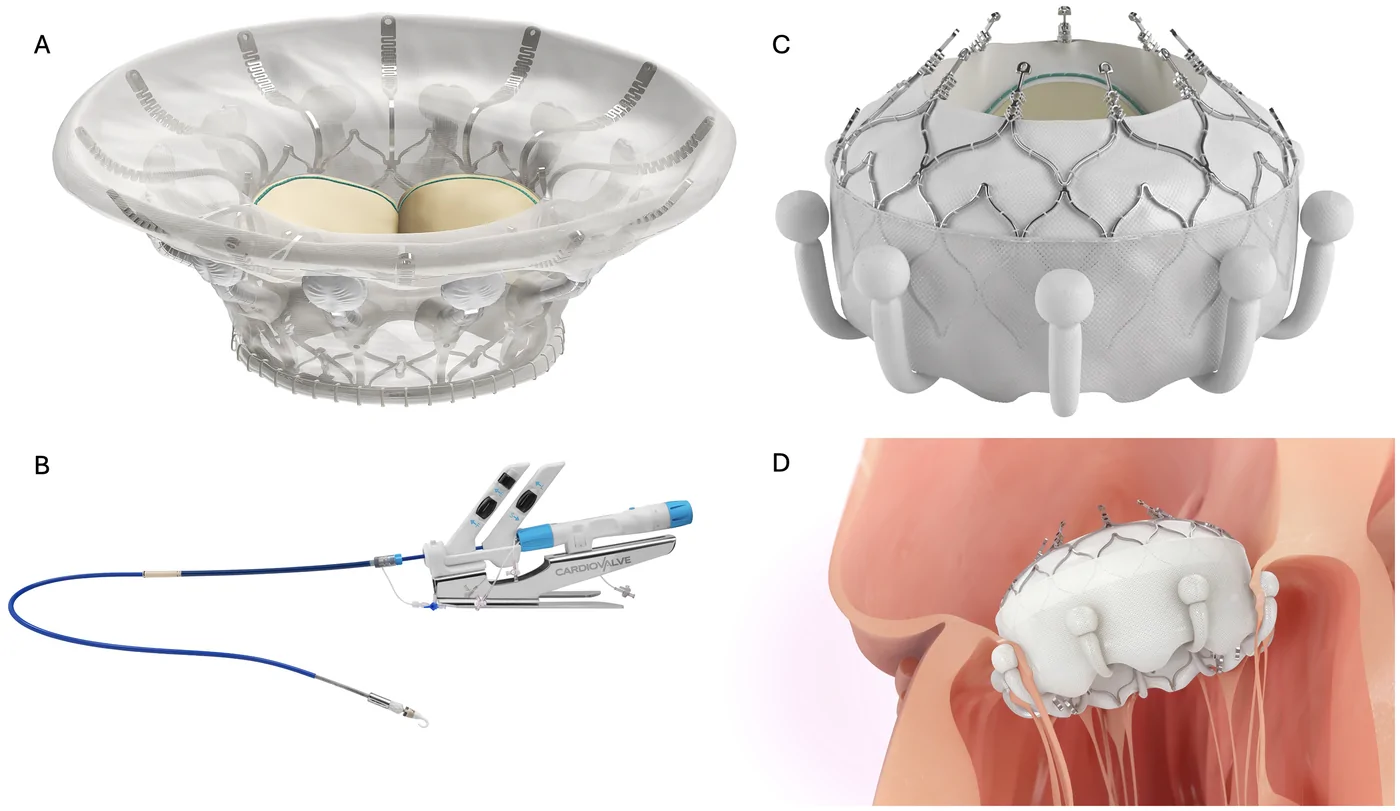
Comparison of both prostheses. Figure shows both prostheses. **(A)** Shows the Cardiovalve (https://cardiovalve.com) prosthesis (Venus MedTech Hangzhou Inc., China) and **(B)** its delivery system. **(C)** Shows the EVOQUE (https://www.edwards.com/healthcare-professionals/products-services/transcatheter-mitral-tricuspid-technologies/evoque-tricuspid-valve-replacement-system) prosthesis (Edwards Lifesciences, Irvine, California, U.S.) with its unique anchoring mechanism in **(D)**.

Transcatheter Tricuspid Valve Replacement (TTVR) has emerged as a treatment option for patients with severe or higher-grade tricuspid regurgitation (TR) who are deemed inoperable by heart team consensus and whose anatomy is sub-optimal for tricuspid transcatheter edge-to-edge repair (T-TEER) (1, 2). Different TTVR devices are available, and recent studies have shown their procedural and clinical success (1, 3–5). The EVOQUE prosthesis (Edwards Lifesciences, Irvine, California, U.S.; see Figure 1) is currently the only one with U.S. Food and Drug Administration (FDA) and CE (Conformité Européenne) mark approval. The TRISCEND and the TRISCEND II trial demonstrated its safety and efficacy (5, 6). The investigators were able to show substantial improvement in patient's symptoms, function and quality of life at 30 days, 6 months and 1 year follow up (4). The Cardiovalve prosthesis (Venus MedTech HangZhou Inc., China; see Figure 1) yet has neither FDA, nor CE mark approval but has applied for the latter one and has acceptance to conduct early feasibility studies in the United States and Europe. The TARGET trial enrolled 150 patients and will evaluate the safety and performance of the Cardiovalve TTVR. Its preliminary results were presented at the 2025 PCR London Valves Conference. At 30 days an acceptable safety profile and effective symptom control were described with a significant reduction of TR grade (97% TR grade ≤ mild) and New York Heart Association (NYHA) functional class (90% ≤ NYHA II).

This study aims to describe procedural and short-term clinical and echocardiographic outcomes of two different TTVR prostheses, as comparative data between devices remain scarce.

## Methods

We performed a retrospective, single-center cohort analysis including all patients who received TTVR with either the Cardiovalve or the EVOQUE prosthesis at the University Heart Center Lübeck in Lübeck, Germany between July 2021 and June 2025. Included were all patients ≥ 18 years of age who had TR grade ≥ 3 and were deemed inoperable or at high surgical risk by decision from the local heart team. Only patients capable of providing informed consent were included. All Cardiovalve procedures were performed within the TARGET study or as compassionate use cases between February 2023 and October 2024. The EVOQUE prosthesis was implanted at our center after CE mark approval between February 2024 and June 2025.

The EVOQUE TTVR prosthesis is a transfemoral, self-expanding transcatheter bioprosthesis, engineered for orthotopic replacement of the native tricuspid valve. The device, which is available in four different sizes (44, 48, 52 and 56 mm), comprises a nitinol frame with large-cell architecture designed to conform to the typically dilated, non-calcified tricuspid anulus. It integrates a ventricular anchoring system (see Figure 1C) that engages the native leaflets and subvalvular apparatus for axial stability, and a circumferential fabric sealing skirt to mitigate paravalvular regurgitation. The valve incorporates bovine pericardial leaflets mounted within the frame to provide immediate hemodynamic competence. Ultrasound guidance for venous access and vascular closure devices were used as standard of care. The prosthesis is delivered via a 28 French (Fr) transfemoral system that allows controlled, stepwise deployment under fluoroscopic and echocardiographic guidance to optimize coaxial alignment and annular positioning prior to final release. The detailed implantation process has been previously described (5). One patient in our cohort underwent EVOQUE implantation after prior T-TEER using a Pascal device Ace. The patient presented with severe TR after T-TEER. No special management of the T-TEER device was necessary, it remained in the anterior-septal commissural position and did not cause relevant interference with the EVOQUE prosthesis. Only minor TR remained after TTVR.

The Cardiovalve TTVR is a transfemoral, self-expanding transcatheter bioprosthesis, developed for orthotopic replacement of native tricuspid (and mitral) valves in patients with severe regurgitation who are at high surgical risk. The device, which is available in three different sizes (45, 50 and 55 mm) consists of a low-profile nitinol frame with a dual-frame configuration and circumferential atrial flange designed to achieve supra-annular sealing, along with multiple ventricular grasping legs that capture and secure the native leaflets to provide active fixation in the absence of annular calcification. Bovine pericardial leaflets are mounted within the frame to ensure immediate hemodynamic competence. The system is delivered via a 37 Fr transfemoral approach using a steerable catheter (see Figure 1B) that permits controlled leaflet capture, positional optimization under fluoroscopic and echocardiographic guidance, and fully retrievable deployment prior to final release. In the Cardiovalve group surgical cutdown of the venous access was carried out in 9 patients (75%) as primarily intended by request from the company. The other 4 procedures were performed with ultrasound guided venous access and vascular closure devices as standard of care. All procedures were performed under general anesthesia.

Data was collected from the patients’ records, including echocardiographic imaging studies at the initial hospital stay and at a scheduled 30-day follow-up. TR was graded on a five-point scale according to the current classification by Hahn et al. (7). All patients additionally received a pre-procedural CT-scan for sizing and procedural planning. The outcomes of this analysis, including intraprocedural and clinical success, were defined in accordance with the Tricuspid Valve Academic Research Consortium (TVARC) definitions for tricuspid regurgitation and trial endpoints (8). Additional outcomes included reduction of TR severity, improvement of NYHA functional class, as well as complications and safety endpoints, such as cardiovascular and all-cause mortality, major vascular complications, stroke, new pacemaker implantation and kidney failure within 30 days.

Approved by the Ethics Committee of the University Hospital Schleswig-Holstein, Campus Lübeck (reference number 2025-242), the study was conducted in accordance with the ethical standards of the hospital and the guidelines laid out in the Declaration of Helsinki. All participants were orally and in writing informed about the study procedure, objectives, and data protection regulations. A specifically designed information sheet with a consent form was used for written consent. Participant anonymity was maintained through the assignment of identification numbers.

### Statistical analysis

Categorical variables are expressed as percentages, continuous variables are expressed as mean. To compare categorical variables, Fisher's exact test was used. For ordinal data, the Mann–Whitney-*U*-test was used for comparisons between independent groups, whereas for paired pre-post comparisons the Wilcoxon signed-rank test was used. Statistical significance was determined with a two-sided *p*-value with a threshold of <0.05. The analysis included all patients on an intention-to-treat basis. All statistical evaluations were performed using SPSS (Statistical Package for Social Sciences) V. 29.0.2.0.

## Results

A total of 25 TTVR procedures were performed at our tertiary center, comprising 12 Cardiovalve and 13 EVOQUE procedures.

The mean age of the patients was 80.6 years, the mean BMI was 24.7 kg/m^2^ and 72% of the patients were female. Overall, 88% of the patients presented with chronic kidney disease, 92% presented with atrial fibrillation, 48% had coronary artery disease and 60% had pulmonary hypertension. Regarding prior valve interventions, 12 (48%) underwent interventional valve repair or replacement: M-TEER was performed in 7 (28%) patients, T-TEER in 1 (4%) and 6 (24%) had undergone transcatheter aortic valve implantation (TAVI). In 12 (48%) of the patients right ventricular leads were present: 11 (44%) had a pacemaker and one (4%) had an implantable cardioverter-defibrillator (ICD). There were no significant differences in baseline characteristics and co-morbidities between the two groups (see Table 1).

**Table 1:** Baseline characteristics.

Patient Characteristics	All (*n* = 25)	Cardiovalve (*n* = 12)	EVOQUE (*n* = 13)	*p*-value
Age, years	80.6 ± 5.3	82.3 ± 2.9	79.2 ± 6.5	.143
Female	18 (72.0)	9 (75.0)	9 (69.2)	1
BMI, kg/m^2^	24.7 ± 4.5	24.7 ± 4.3	24.6 ± 4.9	.966
Arterial hypertension	19 (76.0)	11 (91.6)	8 (61.2)	.16
Diabetes mellitus	5 (20.0)	1 (8.3)	4 (30.8)	.322
Atrial fibrillation	23 (92.0)	12 (100)	11 (84.6)	.48
Chronic obstructive pulmonary disease	1 (4.0)	1 (8.3)	0 (0.0)	1
Coronary artery disease	12 (48.0)	5 (41.6)	7 (53.9)	.695
Prior cardiac surgery	8 (32.0)	3 (25.0)	5 (38.5)	.673
CABG	6 (24.0)	2 (16.6)	4 (30.8)	.645
SAVR	2 (8.0)	1 (8.3)	1 (7.7)	1
Prior valve intervention	12 (48.0)	5 (41.6)	7 (53.9)	.695
TAVI	6 (24.0)	3 (25.0)	3 (23.1)	1
M-TEER	7 (28.0)	3 (25.0)	4 (30.8)	1
T-TEER	1 (4.0)	0 (0.0)	1 (7.7)	1
Pacemaker	10 (40.0)	3 (25.0)	7 (53.9)	.226
ICD	1 (4)	0 (0.0)	1 (7.7)	1
NTproBNP, ng/L	3,714.7 ± 6,372.5	2,414.8 ± 1,621.8	4,914.7 ± 8,692.4	.338
Bilirubin, µmol/L	11.8 ± 5.3	12.7 ± 4.3	11.1 ± 6.1	.458
Hemoglobin, g/dL	11.7 ± 1.8	12.7 ± 1.3	10.9 ± 1.8	.008
Renal disease	22 (88.0)	10 (83.3)	12 (92.3)	.593
GFR < 60 mL/min	16 (64.0)	8 (66.6)	8 (58.3)	1
GFR < 30 mL/min	6 (24.0)	2 (16.6)	4 (30.8)	1
Pulmonary hypertension	15 (60.0)	8 (66.6)	7 (53.9)	.688
sPAP > 40 mmHg	14 (56.0)	8 (66.6)	6 (46.2)	.428
sPAP > 60 mmHg	1 (4.0)	0 (0.0)	1 (7.7)	1
Peripheral oedema	15 (60.0)	4 (33.3)	11 (84.6)	.015
EuroSCORE II, %	5.6 ± 4.9	4.27 ± 2.82	6.83 ± 6.11	.198
STS-Score, %	9.5 ± 5.4	9.9 ± 6.5	9.0 ± 4.3	.68
TRI-SCORE, x/12	4.8 ± 1.55	4.83 ± 1.03	4.77 ± 1.30	.893
GLIDE Score, x/5	2.2 ± 1.08	2.33 ± 1.15	2.00 ± 1.04	.65

Values are mean ± SD, *n* (%) or points. Table shows the baseline characteristics, medical history and the risk score of the patients. There was no significant difference between the two groups for any of the criteria (BMI, body mass index; CABG, coronary artery bypass graft; GFR, glomerular filtration rate; ICD, implantable cardioverter-defibrillator; M-TEER, mitral transcatheter edge-to-edge repair; NTproBNP, N-terminal pro b-type natriuretic peptide; TAVI, transcatheter aortic valve implantation; T-TEER, tricuspid transcatheter edge-to-edge repair; SAVR, surgical aortic valve replacement; sPAP, systolic pulmonary artery pressure; STS, society of thoracic surgeons).

The perioperative risk scores showed an intermediate-risk patient profile with no significant differences between the two groups. The mean EuroScore II was numerically higher in the EVOQUE group (6.83% ± 6.11) compared to the Cardiovalve group (4.27% ± 2.82), but without a statistically significant difference (*p* = 0.198). The mean STS (society of thoracic surgeons) -score and the mean TRI-SCORE were numerically and statistically not different (STS-Score: 9.0% ± 4.3 vs. 9.9% ± 6.5, *p* = 0.680; TRI-SCORE: 4.77 ± 1.30 vs. 4.83 ± 1.03, *p* = 0.893). The mean GLIDE Score (9) in our cohort was 2.2 (±1.08) with no significant difference between the two groups (Cardiovalve: 2.33 ± 1.15; EVOQUE: 2.00 ± 1.04; *p* = 0.65).

**Figure 2:**
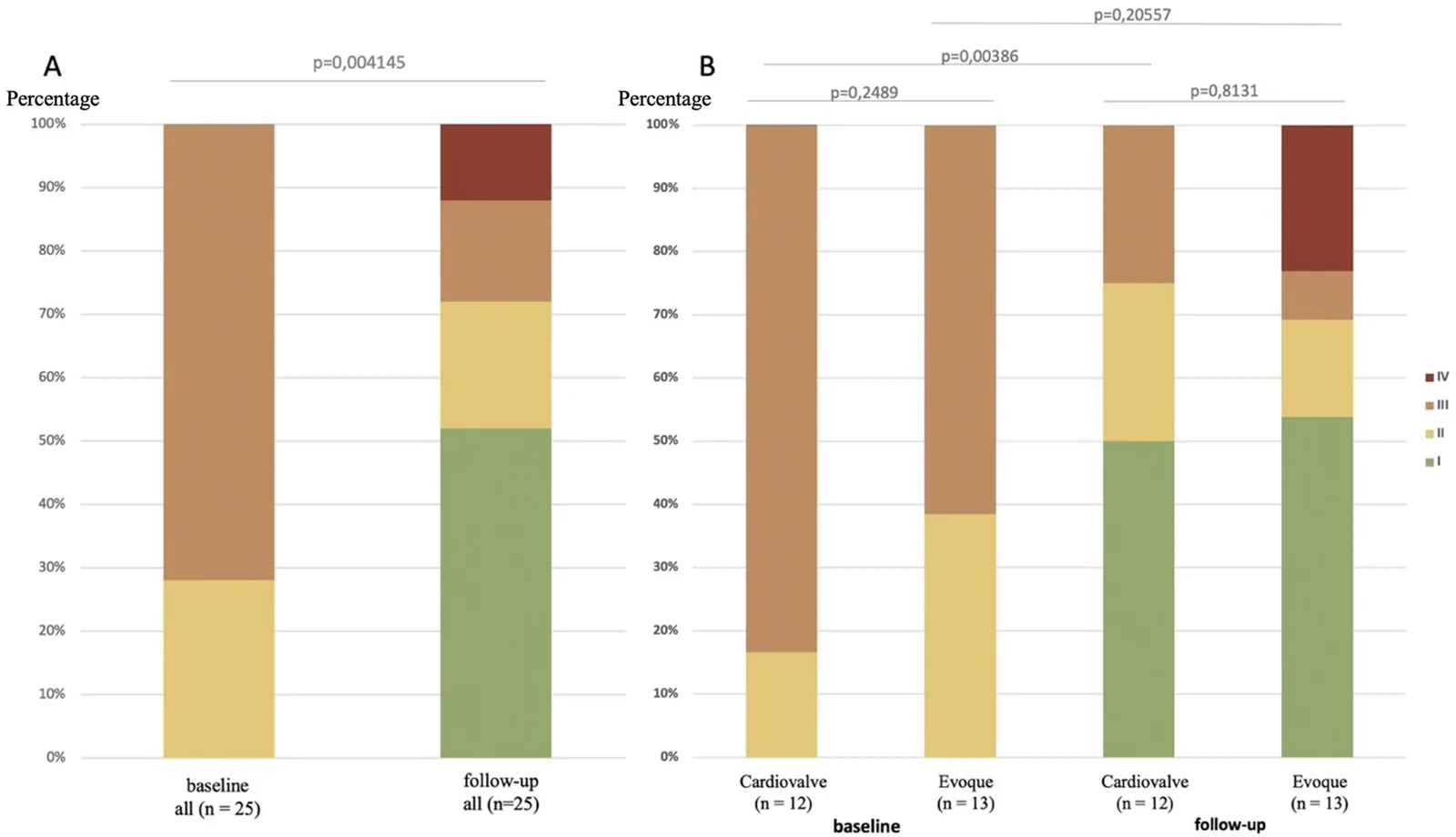
NYHA functional class at baseline and at 30-day follow-up. **(A)** Shows NYHA functional class of all patients at baseline and at 30-day follow-up. At baseline all patients were NYHA functional class II or III, whereas at follow-up most patients were NYHA functional class I, resembling a significant improvement (*p* = .004). **(B)** Shows NYHA functional class of the two groups (Cardiovalve and EVOQUE) at baseline and at 30-day follow-up. There was a significant improvement of the NYHA functional class in the Cardiovalve group but not in the EVOQUE group. (NYHA, New York Heart Association).

Regarding NYHA class, patients presented primarily with NYHA class III (72%) or II (28%). A significant reduction of the average NYHA class could be shown for the entire cohort (median NYHA class at baseline: 3 [IQR, 2–3] vs. median NYHA class at follow-up: 1 [IQR, 1–3]; *p* = 0.004, see Figure 2A), with 18 patients (72%) being NYHA class II or lower at 30-day follow-up. There was no significant difference in NYHA class between the two groups at baseline (*p* = 0.249) and after 30 days (*p* = 0.813). Within the groups a significant reduction of the median NYHA class could be observed in the Cardiovalve group (3 [IQR, 3–3] vs. 2 [IQR, 1–3]; *p* = 0.004) with 9 patients (75.0%) being NYHA ≤ 2 at 30-days follow-up, but not for the EVOQUE group (3 [IQR, 2–3] vs. 1 [IQR, 1–3]; *p* = 0.206) with 9 patients (69.2%) being NYHA ≤ 2 at 30-days follow-up, as three patients in the EVOQUE group were NYHA class IV at follow-up (see Figure 2B).

**Figure 3:**
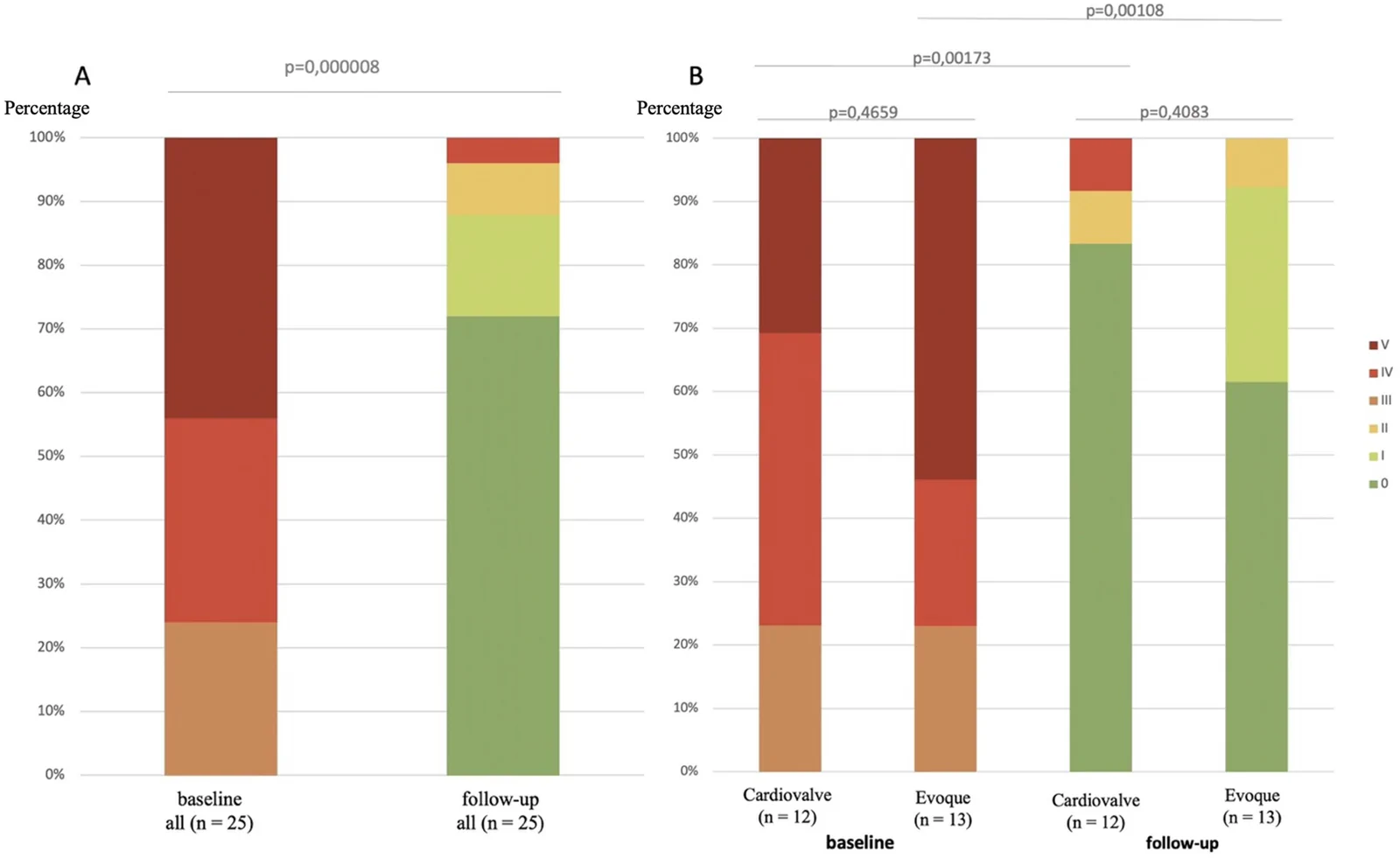
TR grade at baseline and at follow-up. **(A)** Shows TR grade of all patients at baseline and at 30-day follow-up. At baseline all patients had TR grade ≥ 3, whereas at follow-up all except one patient had TR grade ≤ 2 (*p* < .001). **(B)** Shows TR grade of the two groups (Cardiovalve and EVOQUE) at baseline and at 30-day follow-up. There was a significant reduction of the TR grade in both groups (TR, tricuspid regurgitation).

All patients (100%) had TR grade III or higher at baseline. Most (11; 44.0%) presented with TR grade V, followed by 8 patients (32.0%) with TR grade IV and 6 patients (24.0%) with TR grade III. All except one patient (96%) had TR grade II or lower at 30 days follow-up (4.20 vs. 0.48; *p* < 0.001, see Figure 3A). There was no significant difference between the Cardiovalve and the EVOQUE group at baseline and after 30 days (4.08 vs. 4.31; *p* = 0.466 and 0.50 vs. 0.46; *p* = 0.408). Within each group a significant reduction of the TR grade could be observed for both, the Cardiovalve (4.08 vs. 0.50; *p* = 0.002), and the EVOQUE (4.31 vs. 0.46; *p* = 0.001) group (see Figure 3B). One patient in the Cardiovalve group who initially presented with TR grade V remained TR grade IV due to prosthesis dislocation within 48 h and died after 2 months due to decompensated right heart failure.

In the Cardiovalve group the 55 mm prosthesis was implanted in 9 cases (75.0%) and the 50 mm prosthesis in 3 cases (25.0%). In the EVOQUE group the 52 mm prosthesis was the most implanted size (8 cases, 61.5%), followed by size 56 mm and 44 mm which two cases each (15.4%) and the 48 mm prosthesis was implanted in one patient (7.7%).

The average procedure time was 131.56 min with a numerical, but no statistically significant difference between the two devices (148.75 min. for Cardiovalve vs. 115.69 min. for EVOQUE; *p* = 0.0574, see Table 2). It should be noted however, that surgical cut down of the venous access was performed in 75% of the patients in the Cardiovalve group but not in the EVOQUE group.

**Table 2:** Intra- and postprocedural data.

Procedural Data	All (*n* = 25)	Cardiovalve (*n* = 12)	EVOQUE (*n* = 13)	*p*-value
Procedure time, min	131.6 ± 43.8	148.8 ± 43.8	115.7 ± 38.8	.057
Fluoroscopy time, min	28.5 ± 14.8	34.0 ± 19.1	23.4 ± 7.0	.075
Dose area product, cGy/cm^2^	3,366.8 ± 3,481.1	3,243.3 ± 3,407.4	3,480.8 ± 3,683.0	.869
Intraprocedural success	25 (100)	12 (100)	13 (100)	1
Length of hospital stay, days	13.1 ± 11.1	11.7 ± 3.4	14.4 ± 15.3	.553
Days on ICU, days	2.8 ± 4.9	1.1 ± 0.3	4.3 ± 6.5	.099

Values are mean ± SD or *n* (%). Table shows the procedural and postprocedural data of the two device groups. For neither of the criteria a significant difference between the two groups was present (ICU, intensive care unit).

Regarding the echocardiographic variables, the only two differences were a larger vena contracta at baseline in the Cardiovalve group (18.45 vs. 13.42 mm; *p* = 0.029) and a better left ventricular ejection fraction in the Cardiovalve group at both, baseline (57.1% vs. 50.1%; *p* = 0.009) and at 30-day follow-up (57.2% vs. 50.6%; *p* = 0.008). There was no difference between the Cardiovalve and EVOQUE prostheses in right ventricular function or severity of TR at baseline and follow-up (see Table 3).

**Table 3:** Echocardiographic measurements at baseline and at follow-up.

Echocardiographic Measurements	Baseline			Follow-up		
	Cardiovalve (*n* = 12)	EVOQUE (*n* = 13)	*p*-value	Cardiovalve (*n* = 12)	EVOQUE (*n* = 13)	*p*-value
Vena contracta, mm	18.5 ± 5.8	13.4 ± 4.5	.029	1.6 ± 3.9	1.3 ± 1.2	.801
Regurgitation volume, mL	67.4 ± 17.4	67.2 ± 35.4	.982	7.5 ± 19.0	1.5 ± 3.8	.278
EROA, mm^2^	79.3 ± 29.0	89.9 ± 56.6	.57	9.1 ± 23.3	0.02 ± 0.04	.173
sPAP, mmHg	30.6 ± 10.7	29.8 ± 12.4	.862	25.0 ± 3.6	30.1 ± 11.8	.492
VCI diameter, mm	28.1 ± 8.5	25.9 ± 7.3	.508	23.9 ± 5.6	21.8 ± 5.6	.386
TVG, mmHg	1.0 ± 0	1.1 ± 0.2	.331	2.7 ± 0.5	3.1 ± 1.1	.263
RA volume, mL/m^2^	86.3 ± 32.4	86.2 ± 34.6	.992	78.3 ± 23.1	72.2 ± 31.1	.597
TAPSE, mm	17.2 ± 4.3	16.9 ± 5.3	.901	14.5 ± 2.6	12.0 ± 3.3	.061
RV basal diameter, mm	52.3 ± 8.3	49.5 ± 6.2	.334	50.9 ± 7.4	47.4 ± 9.3	.348
RV-FAC, %	37.8 ± 6.6	34.8 ± 6.0	.257	33.3 ± 5.4	29.3 ± 6.5	.115
LV-EF, %	57.1 ± 7.5	50.1 ± 4.5	.009	57.2 ± 6.9	50.6 ± 3.9	.008
MR grade > 1	2 (16.9)	2 (15.4)	1	2 (16.7)	3 (23.0)	1

Values are mean ± SD or *n* (%). Table shows the echocardiographic measurements at baseline and at 30-day follow-up for both, the Cardiovalve and the EVOQUE groups. The only significant differences were the vena contracta at baseline and the LV-EF at baseline and at follow-up. (EROA, effective regurgitation orifice area; FAC, fractional area change; MR, mitral regurgitation; RA, right atrium; RV, right ventricle; sPAP, systolich pulmonary artery pressure; TTVR, transcatheter tricuspid valve regurgitation; TVG, transvalvular gradient).

No difference in pre-procedural right heart catheterization measurements was observed between the two groups (see Table 4).

**Table 4:** Right heart catheterization measurements.

RHC measurements	All (*n* = 25)	Cardiovalve (*n* = 12)	EVOQUE (*n* = 13)	*p*-value
mPAP, mmHg	24.4 ± 6.0	24.2 ± 6.2	24.7 ± 6.1	.833
PCWP, mmHg	17.1 ± 6.3	17.1 ± 6.2	17.1 ± 6.4	.998
CO (Fick), L/min	3.2 ± 1.5	3.4 ± 1.6	3.2 ± 1.4	.77
CO (thermodilution), L/min	3.6 ± 1.3	3.4 ± 0.7	3.8 ± 1.6	.537
CI, L/min/m^2^	2.2 ± 0.7	2.2 ± 0.6	2.2 ± 0.9	.921
SaO_2_, %	96.0 ± 2.4	96.0 ± 2.7	95.9 ± 2.1	.933
SvO_2_, %	57.7 ± 14.0	58.3 ± 14.9	57.1 ± 13.6	.843
TPG, mmHg	7.6 ± 4.8	7.3 ± 3.7	7.8 ± 5.8	.787
RAP, mmHg	12.9 ± 4.9	12.2 ± 4.5	13.5 ± 5.3	.496
PVR, WU	2.0 ± 1.2	2.0 ± 1.2	2.0 ± 1.2	.904

Values are mean ± SD. Table shows the measured parameters during pre-procedural right heart catheterization. No significant difference between the two device groups could be detected (CI, cardiac index; CO, cardiac output; mPAP, mean pulmonary artery pressure; PCWP, pulmonary capillary wedge pressure; PVR, pulmonary vascular resistance; RAP, right atrial pressure; SaO_2_, arterial oxygen saturation; SvO_2_, venous oxygen saturation; TPG, transpulmonary pressure gradient).

Intraprocedural and clinical success, as defined according to the TVARC criteria, was 100.0% (25/25) and 84.0% (21/25), respectively. There were two cases in each group who failed clinical success. In the Cardiovalve group one patient, for whom surgical cutdown of the vein was performed, had a major vascular complication (defined according to TVARC as ≥3) with the need for subsequent blood transfusion. No surgical intervention was necessary. The other patient was found to have prosthesis dislocation due to leaflet-detachment of the posterior leaflet, visible in the discharge echocardiographic examination and residual TR grade 4 at 30-day follow-up. In the EVOQUE group one patient had tricuspid valve stenosis (mean pressure gradient of 7 mmHg) in the echocardiographic examination and another patient needed cardiopulmonary resuscitation on the first postoperative day due to third grade atrio-ventricular block. The patient received a CIED, and no further events were documented.

At least one complication occurred in 7 out of the 25 patients (28.0%, see Table 5). The complication rates between the two groups showed no statistically significant difference (Cardiovalve: 4 [33.3%] vs. EVOQUE: 3 [23.1%]; *p* = 0.67, OR = 1.67, 95% CI: 0.28–9.84). One patient in each group developed third grade atrioventricular block, resulting in the need for pacemaker implantation (2/25; 8.0%; for pacemaker naive patients 2/14; 14.3%). There was no case of post-procedural device malfunction or lead jailing of pre-existing pacemakers.

**Table 5:** Major adverse events and clinical safety endpoints at 30 days.

Endpoints	All (*n* = 25)	Cardiovalve (*n* = 12)	EVOQUE (*n* = 13)	*p*-value
Intraprocedural success	25 (100)	12 (100)	13 (100)	1
Clinical success	21 (84.0)	10 (83.3)	11 (84.6)	1
Cardiovascular mortality	0 (0.0)	0 (0.0)	0 (0.0)	1
All-cause mortality	0 (0.0)	0 (0.0)	0 (0.0)	1
Stroke	0 (0.0)	0 (0.0)	0 (0.0)	1
Renal failure	0 (0.0)	0 (0.0)	0 (0.0)	1
Cardiac surgery	0 (0.0)	0 (0.0)	0 (0.0)	1
Major vascular complications	1 (4.0)	1 (8.3)	0 (0.0)	.48
Bleeding	1 (4.0)	1 (8.3)	0 (0.0)	.48
Arteriovenous fistula	0 (0.0)	0 (0.0)	0 (0.0)	1
Infection	0 (0.0)	0 (0.0)	0 (0.0)	1
Thrombosis	0 (0.0)	0 (0.0)	0 (0.0)	1
Minor vascular complications^a^	5 (20.0)	4 (33.3)	1 (7.7)	.16
Bleeding	2 (8.0)	2 (16.7)	0 (0.0)	.22
Arteriovenous fistula	1 (4.0)	0 (0.0)	1 (7.7)	1
Infection	1 (4.0)	1 (8.3)	0 (0.0)	.48
Thrombosis	1 (4.0)	1 (8.3)	0 (0.0)	.48
New CIED (*n* = 25)	2 (8.0)	1 (8.3)	1 (7.7)	1
New CIED (pacemaker-naïve, *n* = 14)	2 (14.3)	1 (11.1)	1 (20.0)	1
Device-related complication	4 (16.0)	2 (16.7)	2 (15.4)	1
Dislocation/detachment	2 (8.0)	2 (16.7)	0 (0.0)	.22
Stenosis	1 (4.0)	0 (0.0)	1 (7.7)	1
Leaflet thrombosis	1 (4.0)	0 (0.0)	1 (7.7)	1
Major	0 (0.0)	0 (0.0)	0 (0.0)	1
Minor	1 (4.0)	0 (0.0)	1 (7.7)	1

Values are *n* (%); Table shows all complications that occurred. Complications were defined according to the TVARC criteria. The most common complications were vascular complications, occurring overall in 24% of the cases. There was no difference in complication rate between the two device groups. There was no case of cardiovascular or all-cause mortality (CIED, cardiac implantable electronic device; TVARC, tricuspid valve academic research consortium).

a Minor vascular complications were not counted towards the overall complication rate.

There was no case of cardiovascular or overall mortality within the 30-day follow-up. Also, no patient needed surgical intervention.

## Discussion

This single-center retrospective analysis is the first to compare intraprocedural and clinical outcomes of 25 consecutive patients who underwent TTVR with either the Cardiovalve or the EVOQUE prosthesis. Our pilot findings allow for a more differentiated use of TTVR prostheses, based on device performance and patient characteristics. The major findings of this study are:

- Implantation of either device led to a significant reduction of TR grade severity.
- Intraprocedural success was 100% and clinical success was 84% for both devices.
- No significant difference in complication rate between the two devices could be observed.
- Mortality rate at 30-day follow-up was 0%.

The calculated risk scores (EuroSCORE II, STS-Score and TRI-SCORE) showed an intermediate risk for the overall cohort and no significant differences between the two groups. The risk scores of our cohort are comparable to that of the TRISCEND and TRISCEND II trials, all reporting groups at intermediate perioperative risk (EuroSCORE II: 5.6% ± 4.9 vs. 5.1% and 5.4%; STS-Score: 9.5% ± 5.4 vs. 10.0% and 9.6%) (5, 6).

Intraprocedural success as defined by the TVARC criteria was 100% in both groups, showing that both prostheses can be safely and effectively implanted. There was a significant reduction in TR grade at 30-day follow-up for both, the Cardiovalve (*p* = 0.002) and the EVOQUE (*p* = 0.001) group with no significant difference between the groups (*p* = 0.408). Overall, 22 patients (88.0%) had TR grade I or less at 30-day follow-up. This shows that TTVR with either prosthesis is highly effective in the treatment of TR. However, the 1-year results of the TRISCEND and TRISCEND II trial showed a slightly higher rate of TR reduction to TR grade I or less of 97.6% and 95.3%, respectively (5, 6). These large landmark studies were prospective randomized controlled trials, whereas we describe a retrospective real world patient cohort. Some of our patients would not have met the inclusion criteria of the TRISCEND and TRISCEND II study protocols. Our consecutive series supports the generalizability of data derived from selected clinical trial patients to routine clinical practice.

In our cohort TTVR was selected for patients with severe or higher-grade TR whose anatomy was not optimal for T-TEER, as assessed with the help of the GLIDE-Score, or in the context of ongoing studies. The average GLIDE Score was >2 for the overall cohort, showing that T-TEER would likely have led to suboptimal results (9). Nonetheless, the functional improvements in our cohort were in fact better than the results of T-TEER in large registries. The bRIGHT registry, which investigated T-TEER with the TriClip (Abbott, Chicago, Illinois, U.S.), reported a TR reduction to grade II or less in 81% at 1 year (10). In the PASTE registry, which investigated T-TEER with the Pascal ACE system (Edwards Lifesciences, Irvine, California, U.S.), reported a TR reduction to grade II or less in 83% at 1 year (11).

A significant reduction in functional NYHA class could be shown in the Cardiovalve group (*p* = 0.004), but not in the EVOQUE group (*p* = 0.206, see Figure 2B). This is mainly driven by 3 patients in the EVOQUE group who were functional NYHA class IV at follow-up. Although those 3 patients showed good procedural and echocardiographic results with an initial TR grade reduction to grade I or less, all three had significant co-morbidities which led to short-term mortality (>30 days, <90 days), all caused by non-cardiovascular diseases.

The baseline echocardiographic measurements of our cohort are also comparable to the TRISCEND and TRISCEND II studies (TR grade ≥ severe: 100% vs. 88% and 100%; LV-EF: 53% vs. 55% and 54%; TAPSE: 17 mm vs. 15 mm and 16 mm), as well as to the recently published TRIPLACE registry (TR grade ≥ severe: 100%; LV-EF: 56%; TAPSE 17 mm) (5, 6, 12). The fact that our patient collective, which represents a real-world cohort, does not only have similar risk scores, but also similar echocardiographic baseline parameters, allows for excellent comparability to the large trials that were prospectively conducted. Our findings, which are also well comparable to the aforementioned trials, support their results in a real-world cohort.

The presence of CIED remains one of the major challenges in TTVR. In our cohort the patients with RV leads and residual postprocedural TR showed to have paravalvular leakage primarily at the location of the leads. This is analogous to the recently published results of the TRIPLACE registry (13). Out of 395 patients, 104 had RV leads in place. The registry reported higher rates of moderate or greater paravalvular leak in those patients, compared to patients who did not have CIEDs (17.1% vs. 7.1%; *p* < 0.017). Not only may the presence of RV leads, interfere with intraprocedural imaging, as it was demonstrated in specific cases (14), but also there is the risk of damaging the leads during the implantation (15, 16). In our cohort there was no case of CIED malfunction after TTVR. It remains unclear how to proceed in case of CIED infection after TTVR. Until now this problem remains a topic of major discussions. Singular cases have shown the successful extraction of RV leads after TTVR (17), but official recommendations are lacking.

In our study two patients (8.0% of the overall cohort and 14.3% of pacemaker-naïve patients) required the implantation of a new permanent pacemaker. Compared to other trials this is a relatively low number. Angellotti et al. reported an implantation rate of 18.9% in pacemaker-naïve patients after TTVR and in the TRISCEND II trial 27.9% of pacemaker-naive patients required new permanent pacemaker implantation (3, 6). The patient in the Cardiovalve group had a pre-existing conduction disturbance in the form of persistent AF and bifascicular block. Angelotti et al. identified pre-existing conduction disturbances, such as right or left bundle branch block or atrioventricular block, as the only independent risk factor for postprocedural pacemaker implantation (3).

As shown in Table 5, most vascular complications were minor, and the most common vascular complication was bleeding at the venous access site. The Cardiovalve delivery system has a 37 Fr diameter and the EVOQUE delivery system has 28 Fr. This is substantially larger than for other procedures (e.g., T-TEER 24 Fr). The fact that the risk for vascular complications rises with an increase in sheath size is well known and has been demonstrated in several studies (18–20). In our cohort, bleeding occurred only in the Cardiovalve group which uses a larger delivery system than that of the EVOQUE prosthesis (37 vs. 28 Fr). Additionally, the vascular complication rate may have been influenced by the surgical cut down of the vein, which was performed in 75% of the Cardiovalve cases. The delivery sheath was altered by the manufacturer, allowing for easier venous access and nihilating the need for surgical cut down for the last 25% of the procedures.

In the Cardiovalve group there were two cases of device dislocation. Prosthesis dislocation or migration is rare but needs to be accounted of as it bears potential for serious complications. In the TRISCEND trial one patient underwent valve-in-valve intervention due to a partially detached valve and in the TRIPLACE registry there were two reported cases of device migration (5, 12). In the EVOQUE group there was one case of tricuspid stenosis and one case of leaflet thrombosis. The patient with stenosis after EVOQUE implantation had a mean transvalvular pressure gradient of 7 mmHg at the second postoperative day. This is a rare finding and is not reflected in current literature on TTVR. In an additional postinterventional TEE, an acute thrombosis or leaflet malfunction was ruled out, as well as a underexpansion of the prosthesis. We therefore suspect a hyperdynamic situation to have be the cause for the high gradient. In the TRISCEND II trial there was no case of tricuspid stenosis reported at 1-year follow-up (4). In our study a single patient with early leaflet thrombosis after TTVR with the EVOQUE prosthesis was an incidental finding during discharge echocardiography with an elevated mean gradient but without any clinical correlation at 30-day follow-up. Subclinical hypoattenuated leaflet thickening (HALT) is a common occurrence after TTVR, with a frequency of up to 24% (21). Most cases are subclinical and do not lead to hemodynamic compromise. However, in the rare occurrence of clinically significant cases (2%–4%), symptomatic valve function with concomitant right heart failure may occur. Therefore, close monitoring of these patients is advised and if HALT becomes clinically significant, anticoagulation has been shown to be effective in treatment (1, 21).

There were no cases of cardiovascular or overall mortality within the 30-day follow-up which is different to the TRISCEND and TRISCEND II trials, which reported a cardiovascular mortality of 1.7% and 3.1% at 30 days. However, two patients in the Cardiovalve group and four patients in the EVOQUE group died within 90 days after the procedure due to non-cardiovascular reasons.

### Limitations

This study was conducted as a retrospective single-center cohort analysis. It is therefore purely observational and bares the risk of information bias and confounding. The sample size of the analysis was small, thereby limiting the generalizability of the conclusions. The procedures were carried out at two different time slots. First the Cardiovalve procedures were carried out between July 2021 and October 2024, followed by the EVOQUE procedures between February 2024 and June 2025. A potential learning curve for the operators cannot be deducted from the data but might have had an impact on the numerically lower complication rate in the EVOQUE cohort. The follow-up was limited to 30 days, thereby restricting the informative value of the findings.

## Conclusions

This study demonstrates that both, the Cardiovalve and EVOQUE prosthesis are very effective in reducing TR with a 100% intraprocedural success rate. No significant difference in complication rates, clinical or echocardiographic outcome between the two prostheses could be shown. Both devices led to significant decrease in TR and functional NYHA class.

These findings support the implementation of TTVR procedures in daily clinical practice at specialized centers in suitable patients. Whether this indication will be expanded requires further research in the form of randomized controlled trials. More research is also needed to further examine the difference between the available TTVR prosthesis and to determine specific patient subgroups who benefit from prosthesis selection.

## Impact on daily practice

These findings support integrating TTVR into routine care at experienced centers, given the high procedural success and consistent reduction in tricuspid regurgitation with both, the Cardiovalve and the EVOQUE system. Clinicians can anticipate significant reduction in TR grade and meaningful symptomatic improvement, reflected by reductions in NYHA class. Careful patient selection remains essential, particularly in those with advanced disease or prior interventions. Future evidence from randomized trials is needed before expanding indications to patients with potentially limited TR reduction after T-TEER and to refine prosthesis-specific selection strategies.

## Data Availability

The raw data supporting the conclusions of this article will be made available by the authors upon special request.
